# A Novel Vascular Multi‐Etiology Model of Alzheimer's Disease‐Related Dementias (ADRD)

**DOI:** 10.1002/alz70855_098657

**Published:** 2025-12-23

**Authors:** Kareem Abdelsaid, Yasir Abdul, Sarah Jamil, Weiguo Li, Adviye Ergul

**Affiliations:** ^1^ Medical University of South Carolina, Charleston, SC, USA; ^2^ Ralph H. Johnson VA Health Care System, Charleston, SC, USA

## Abstract

**Background:**

Vascular Contributions to Cognitive Impairment and Dementia (VCID) is a leading cause of ADRD, which affects over 6.5 million Americans. Approximately 40% of all dementia patients are type 2 diabetic (T2D). Studies have shown that individuals with T2D are at a 2 to 4‐times increased risk of cognitive impairment. Limitations in the current preclinical models that investigate underlying mechanisms mostly rely on chronic hypoperfusion‐mediated neuroinflammation mechanisms forsaking vascular ones. This study aimed to develop a novel multi‐etiology vascular model of VCID/ADRD in control and diabetic animals that would be more clinically related to ADRD patients.

**Methods:**

Control and diabetic male Wistar rats were subjected to sham or a novel multi‐etiology VCID/ADRD model of microemboli (ME) injection followed by unilateral common carotid artery occlusion (UCCAO) within 15 minutes after injection. Animals were evaluated by various behavioral tests till termination at week 22. Behavioral tests included Novel Object Recognition and open‐field tests. Hematoxylin/Eosin and Luxol‐fast blue (LFB) staining were used to assess brain pathologies. Immunoblotting(IB) was used to assess the senescence and hypoxia markers. MarkVCID plasma biomarkers were assessed using mesoscale technology.

**Results:**

H&E staining revealed that tissue damage in the striatum was greater with a surgery and diabetes significance of *p=*0.0012 and *p=*0.0334, respectively. Furthermore, the corpus callosum myelination score was significantly lower in the diabetic UCCAO+ME group (0.78) compared to diabetic (1.70) and control sham (2.46) groups. Animals also displayed anxiety‐like behavior. Our novel z‐scoring holistic behavior analysis method revealed that the UCCAO+ME diabetic group had a z‐score of (‐1.15), compared to (0.08) for Control UCCAO+ME and (0.549) for diabetic sham groups. Moreover, IB revealed a significant increase in hypoxia marker HIF1α (2.75 folds) and senescence marker p21(3.5 folds) in the cortex homogenates. While the control UCCAO+ME group showed lower plasma levels of GFAP and Neurofilament L after injury, indicative of an adaptive repair response, the diabetic UCCAO+ME failed to do so.

**Conclusion:**

Further characterization of this clinically relevant promising multi‐etiology model may identify novel mechanisms contributing to the onset and progression of VCID/ADRD.

